# Real-Time Vehicle Make and Model Recognition with the Residual SqueezeNet Architecture

**DOI:** 10.3390/s19050982

**Published:** 2019-02-26

**Authors:** Hyo Jong Lee, Ihsan Ullah, Weiguo Wan, Yongbin Gao, Zhijun Fang

**Affiliations:** 1 Division of Computer Science and Engineering, CAIIT, Chonbuk National University, Jeonju 54896, Korea; wanwgplus@gmail.com; 2 Department of Robotics Engineering, Daegu Gyeongbuk Institute of Science and Technology, Daegu 42988, Korea; ihsanullah736@gmail.com; 3 School of Electrics and Electronic Engineering, Shanghai University of Engineering Science, Shanghai 201620, China; gaoyongbin@sues.edu.cn (Y.G.); zjfang@foxmail.com (Z.F.)

**Keywords:** vehicle make recognition, deep learning, residual SqueezeNet

## Abstract

Make and model recognition (MMR) of vehicles plays an important role in automatic vision-based systems. This paper proposes a novel deep learning approach for MMR using the SqueezeNet architecture. The frontal views of vehicle images are first extracted and fed into a deep network for training and testing. The SqueezeNet architecture with bypass connections between the Fire modules, a variant of the vanilla SqueezeNet, is employed for this study, which makes our MMR system more efficient. The experimental results on our collected large-scale vehicle datasets indicate that the proposed model achieves 96.3% recognition rate at the rank-1 level with an economical time slice of 108.8 ms. For inference tasks, the deployed deep model requires less than 5 MB of space and thus has a great viability in real-time applications.

## 1. Introduction

In recent years, a plethora of innovative technologies and solutions are bringing intelligent transportation systems (ITSs) closer to reality. ITSs are advanced transportation systems that aim to advance and automate the operation and management of transport systems, thereby improving upon the efficiency and safety of transport. ITSs combine cutting-edge technology such as electronic control and communications with means and facilities of transportation [1]. The development of digital image processing and computer vision techniques offers many advantages in enabling many important ITSs applications and components such as advanced driver-assistance systems (ADASs), automated vehicular surveillance (AVS), traffic and activity monitoring, traffic behavior analysis, traffic management, among others. Vehicle make and model recognition (MMR) is of great interest in these applications, owing to heightened security concerns in ITSs.

**Figure 1:**
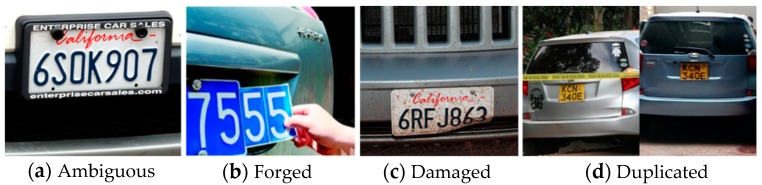
Some cases that cause failure of the license plate recognition-based MMR systems.

Over the years, numerous studies have been conducted to solve different challenges in vehicle detection, identification, and tracking. However, classification of vehicles into fine categories has gained attention only recently, and many challenges remain to be addressed [2,3,4,5]. The traditional vehicle MMR system relies on manual human observation or automated license plate recognition (ALPR) technique, these indirect progresses make the vehicle MMR system hardly meets the real-time constraints. Through manual observation, it is practically difficult to remember and efficiently distinguish between the wide variety of vehicle makes and models; it becomes a laborious and time-consuming task for a human observer to monitor and observe the multitude of screens and record the incoming or outgoing makes and models or to even spot the make and model being looked for. The MMR systems that rely on license plates may suffer from forging, damage, occlusion, etc., as shown in Figure 1. In addition, there are some license-plates that can be ambiguous in terms of interpreting letters (e.g., between “0” and “O”) or license types. Moreover, in some areas, it may not be required to bear the license plate at the front or the rear. If the ALPR system is not equipped to check for license plates at both (front and rear) ends of the vehicle, it could fail. Consequently, when ALPR systems fail to correctly read the detected license plates due to the above issues, the wrong make-model information could be retrieved from the license-plates registry or database.

To overcome the aforementioned shortcomings in traditional vehicle identification systems, automated vehicle MMR techniques have become critical. The make and model of the vehicle recognized by the MMR system can be cross-checked with the license-plate registry to check for any fraud. In this dual secure way, vision-based automated MMR techniques can augment traditional ALPR-based vehicle classification systems to further enhance security.

Abdel Maseeh et al. [6] proposed an approach to address this specific problem by combining global and local information and utilizing discriminative information labelled by a human expert. They validated their approach through experiments on recognizing the make and model of sedan cars from single view images. Jang and Turk [7] demonstrated a car recognition application based on the SURF feature descriptor algorithm, which fuses bag-of-words and structural verification techniques. Baran et al. [8] presented a smart camera in intelligent transportation systems for the surveillance of vehicles. The smart camera can be used for MMR, ALPR, and color recognition of vehicles. In [9], Santos et al. introduced two car recognition methods, both relying on the analysis of the external features of the car. The first method evaluates the shape of the car’s rear, exploring its dimensions and edges, while the second considers features computed from the car’s rear lights, notably, their orientation, eccentricity, position, angle to the car license plate, and shape contour. Both methods are combined in the proposed automatic car recognition system. Ren et al. [10] proposed a framework to detect a moving vehicle’s make and model using convolutional neural networks (CNNs). Dehghan et al. [11] proposed a CNN-based vehicle make, model, and color recognition system, which is computationally inexpensive and provides state-of-the-art results. Huang et al. [12] investigated fine-grained vehicle recognition using deep CNNs. They localized the vehicle and the corresponding parts with the help of region-based CNNs (RCNNs) and aggregated their features from a set of pre-trained CNNs to train a support vector machine (SVM) classifier. Gao and Lee [13] proposed a local tiled CNN (LTCNN) strategy to alter the weight-sharing scheme of CNN with a local tiled structure, which can provide the translational, rotational, and scale invariance for MMR.

In this study, we focus on addressing the challenges in real time and automated vehicle MMR by utilizing state-of-the-art deep learning-based techniques. Vanilla CNNs have demonstrated impressive performance in vehicle MMR-related tasks. However, long training periods and large memory requirements in deployment environments often constrain the use of such models in real-time applications as well as distributed environments. Thus, in this study, a smaller CNN architecture named SqueezeNet is adopted, which requires much fewer parameters, consequently reducing memory constraints and making it suitable for real-time applications. In addition, compared to vanilla SqueezeNet, the proposed method, using a bypass connection-inspired version of SqueezeNet, demonstrates a 2.1% improvement in recognition accuracy. Further, we demonstrate the effectiveness of a data-clustering approach used in our study that considerably improves the speed of the data preparation and labelling process. The experiment results show that the proposed approach obtains higher recognition accuracy in less time and with fewer memory constraints.

## 2. Background

### 2.1. General Architecture of Vehicle MMR System

**Figure 2:**
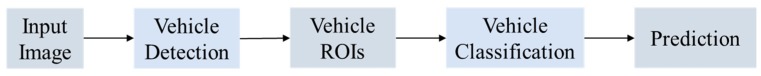
Flowchart of the typical MMR system.

The problem of automated vehicle classification into makes and models is an important task for AVS and other ITSs applications. The general architecture of the vehicle MMR system can be depicted as shown in Figure 2. Most studies first adopt a vehicle detection step that produces regions of interests (ROIs) containing the vehicles’ faces (front), segmented from the background. The vehicle MMR systems then work on these ROIs [14].

The traditional vehicle detection methods can be mainly divided into three categories [15]. One is the edge feature-based method, which first detects possible vehicle candidates from input images, then employing appropriate algorithms for verification [16]. Another method is the color-based method, which utilizes the large variations in vehicle colors [17]. The third commonly-used vehicle detection method involves training a robust vehicle detector through active learning tools such as AdaBoost [18]. Nowadays, the CNNs based vehicle detection methods has been widely applied, such as Fast R-CNN [19], Faster R-CNN [20], and YOLO [21] etc. As for the vehicle recognition, the traditional method is to extract vehicle features such as Haar and SIFT features. Then, the classifiers such as SVM and K-nearest neighbors are learned to classify the vehicle into different models. Because of the variations in the visual appearances and the confusion between certain vehicle types, developing a highly accurate vehicle MMR system is still very challenging using the traditional methods.

### 2.2. Convolutional Neural Networks

**Figure 3:**
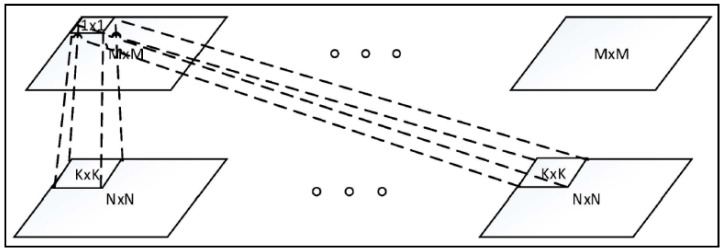
Illustration of one convolutional layer.

Recently, deep networks are increasingly being used to extract discriminative features for vehicle MMR. The deep network concept has been around since 1980, with similar ideas including neural network and backpropagation. The resurgence of interest in deep networks has been brought about by the breakthrough in Restricted Boltzmann Machines (RBM) from Hinton [22]. In the deep networks, there are massive parameters which require large-scale datasets for training. The CNNs are usually adopted to reduce the number of parameters. In addition, many advanced techniques such as dropout, maxout, and max-pooling have been coupled into the CNN structure. Figure 3 shows one convolutional layer. By going deeper into the convolutional networks, CNNs dictate the performance of various applications such as AlexNet [23] and GoogLeNet [24].

Figure 3 illustrates the architecture of one convolutional layer, in which the bottom layer includes several N × N inputs and the top layer includes several M × M convolutional outputs. The convolutional layer conducts convolution operations across the input maps with a K × K filter for each map, resulting in a (N − K + 1) × (N − K + 1) feature map. Here, M = N − K + 1.

### 2.3. SqueezeNet

**Figure 4:**
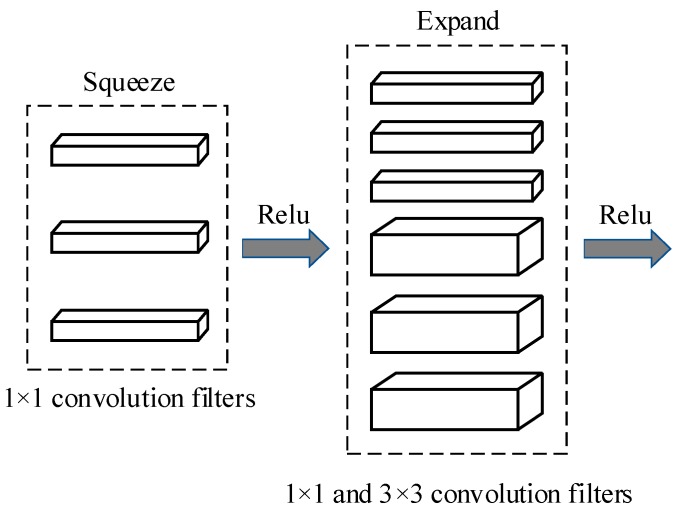
Micro-architectural view: Convolution filters organization in the Fire modules.

The SqueezeNet [25] is a smaller CNN architecture that uses fewer parameters while maintaining competitive accuracy. Several strategies are employed on the CNN basis to design the SqueezeNet: (1) replace 3 × 3 filters with 1 × 1 filters, (2) decrease the number of input channels to 3 × 3 filters, (3) downsample late in the network so that the convolution layers have large activation maps. The SqueezeNet is comprised mainly of Fire modules that are squeeze convolution layers with only 1 × 1 filters. These layers are then fed into an expand layer, which has a mix of 1 × 1 and 3 × 3 convolution filters, as shown in Figure 4.

## 3. Vehicle Data Clustering and Labelling

Deep learning typically benefits from large amounts of data. We create a dataset pool designed specifically for the task of recognition of the vehicle make and model. Date set are collected from currently driven vehicles in Korea, including Korean manufactured and commonly imported vehicles for proposed network. In the following sections, data clustering and labeling are discussed in detail.

### 3.1. Dataset Clustering

In order to train deep networks, a large-scale dataset is required, labelling of which is difficult and tedious. Thus, a cluster method is required for speeding up the labelling process. The appearance of a vehicle will change under varying environmental conditions and also based on market requirements. This makes the vehicle model recognition a challenging task.

**Figure 5:**
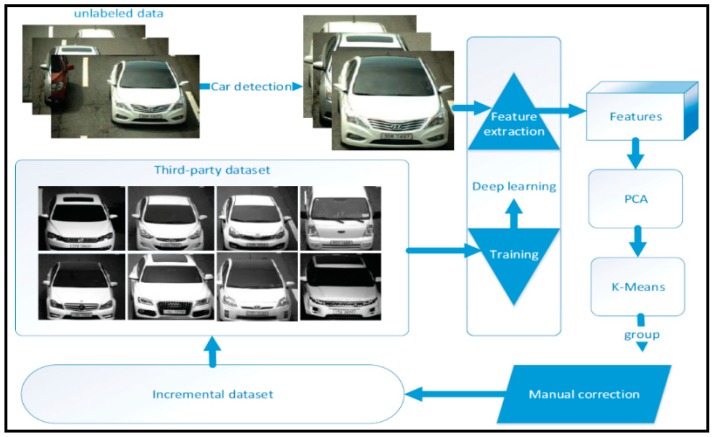
Framework for the proposed car detection and clustering method.

A cluster algorithm aims to differentiate images into groups so that the distance between the different groups is highest. The K-means algorithm is most efficient and suitable for large-scale datasets. However, if we apply the K-means directly on the raw images, the accuracy is compromised because the raw images are not discriminative. This problem will result in burdensome tasks to manually relabel the incorrect vehicle data. In addition, the computational time of this direct clustering strategy was high because the raw images were of large size. In this study, a cluster method that incorporates deep learning is adopted to assist faster labeling of a large-scale vehicle make and model dataset. The cluster method can automatically divide the images into groups and each group is divided into classes. The framework for data clustering is shown in Figure 5 and the detailed steps are as follows:

(1) The vehicles are first detected based on frame difference and symmetrical filter. The frame difference method is applied to images by shifting one image with moderate pixels to generate another image. The difference between these two images is used to detect the vehicle by a symmetrical filter, which makes use of the symmetrical structure of the vehicles.
(2) Then, the discriminative and simple features are extracted based on deep learning before using the K-means algorithm. To use deep learning for feature extraction, we introduce a third-party dataset, which may have a lower number of images and car models. The third-party dataset named as “compcars” [26] is labelled and used for training the deep network. The trained model is used to extract features of the unlabeled vehicles. However, the features are still high-dimension for adopting the K-means algorithm. Thus, we use principal component analysis (PCA) to reduce the dimensions.
(3) Finally, the K-means algorithm is used to cluster the data and assign the group to each data. The manual correction of the wrongly clustered data is performed to get a new dataset. This dataset is added to the third-party dataset to train a more powerful deep network. In this sense, the labelling is performed iteratively. For every iteration, we introduce 100,000 images as incremental data.

**Figure 6:**
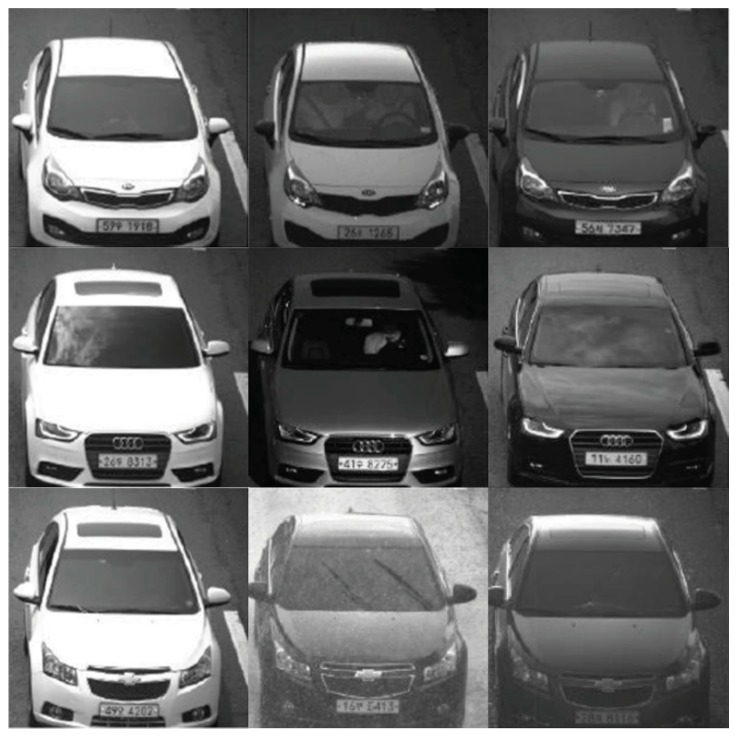
Examples of clustered images.

Our study focuses on the surveillance vehicle data while the “compcars” contain only 44,481 images in 281 different models, which is not enough for practical use. Thus, we collect 291,602 images from the surveillance camera that is set up on an actual street. The data collection time spans one year, and we divide the images into two sets: set-1 (145,800 images) and set-2 (145,802 images). We first use the “compcars” images to train a deep model and then extract features for the set-1. After clustering this set, we use these images to train a new model. The new model is used to cluster the set-2. Figure 6 shows some examples of clustered images. These images contain many kinds of variations such as rainy/sunny, daytime/nighttime, illumination variations, and parts missing due to the car detection failure. As a result, a large-scale dataset containing 291,602 images has been labelled, which consists of 766 models. The accuracy of the cluster of set-2 reached 85%, which means that the second iteration of the cluster reduced the manual label work of 85%. Thus, our cluster sped up the labelling work significantly.

### 3.2. Dataset Labelling

As mentioned earlier, we have labelled 766 models for the 291,602 vehicle images collected. We labelled each vehicle with its respective model name, make and type with its respective class identification. Some of the datasets are shown in Table 1.

## 4. The Proposed MMR System

### 4.1. Residual SqueezeNet Architecture

After vehicle detection and clustering, we train a deep neural network capable of recognizing 766 models of different companies. In this study, a smaller CNN called SqueezeNet [25] is employed, which can achieve performance comparable with other CNN frameworks such as AlexNet while requiring fewer parameters, which is practical in a real-time scenario.

**Figure 7:**
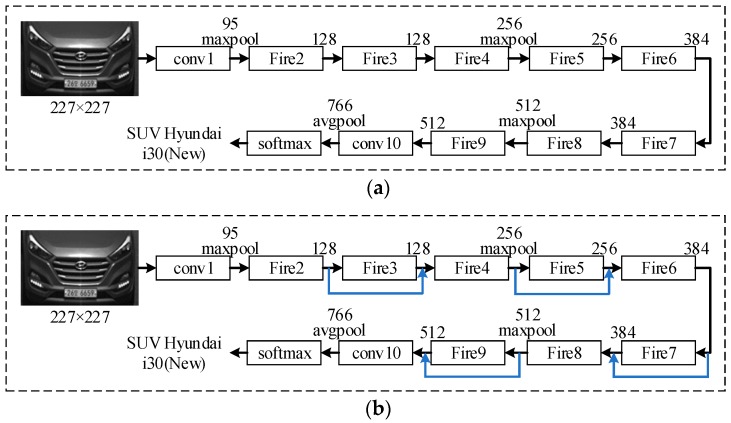
Illustration of SqueezeNet-based MMR system. (**a**) SqueezeNet architecture. (**b**) Our proposed Residual SqueezeNet architecture.

As shown in Figure 7a, the basic SqueezeNet starts with a convolution layer, followed by eight Fire modules, ending with another convolution layer. The number of filters per Fire module is increased gradually from the beginning to the end of the network. The max-pooling with a stride of two is performed after layers conv1, Fire4, Fire8, and conv10. ReLU is adopted as the activations function and Dropout with a ratio of 0.5 is used after the Fire9 module.

**Figure 8:**
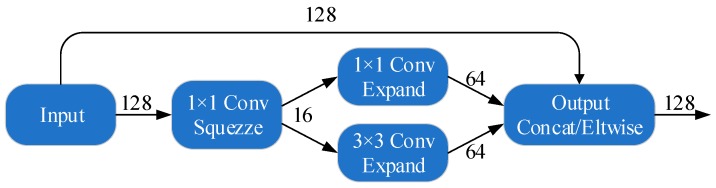
Proposed residual Squeeze Net architecture with simple bypass connections.

To improve the recognition accuracy, a modified SqueezeNet is designed by adding some simple bypass connections to the SqueezeNet between some Fire modules. In our simple bypass architecture, bypass connections are added around Fire modules 3, 5, 7, and 9, requiring these modules to learn a residual function between input and output as shown in Figure 7b. As in ResNet, to implement a bypass connection around Fire3, we set the input to Fire4 equal to the output of Fire2 + output of Fire3, where the + operator is an element-wise addition, as shown in the Figure 8. This changes the regularization applied to the parameters of these Fire modules and, as per ResNet, can improve the final accuracy and/or trainability of the full model.

During training process, the weight parameters are randomly initialized with a Gaussian distribution. The learning rate is initially set as 0.01 and decreases with step size of 10. In addition, the RMSprop is applied as the optimizer.

### 4.2. Hardware and Software

We used a GeForce GTX 1080 in our setup to benefit from faster training times in deep learning frameworks with the support of Cuda and cuDNN. The CPU used is an Intel(R) Core (TM) i7-4790 CPU with eight cores operating at 3.60 GHz.

**Figure 9:**
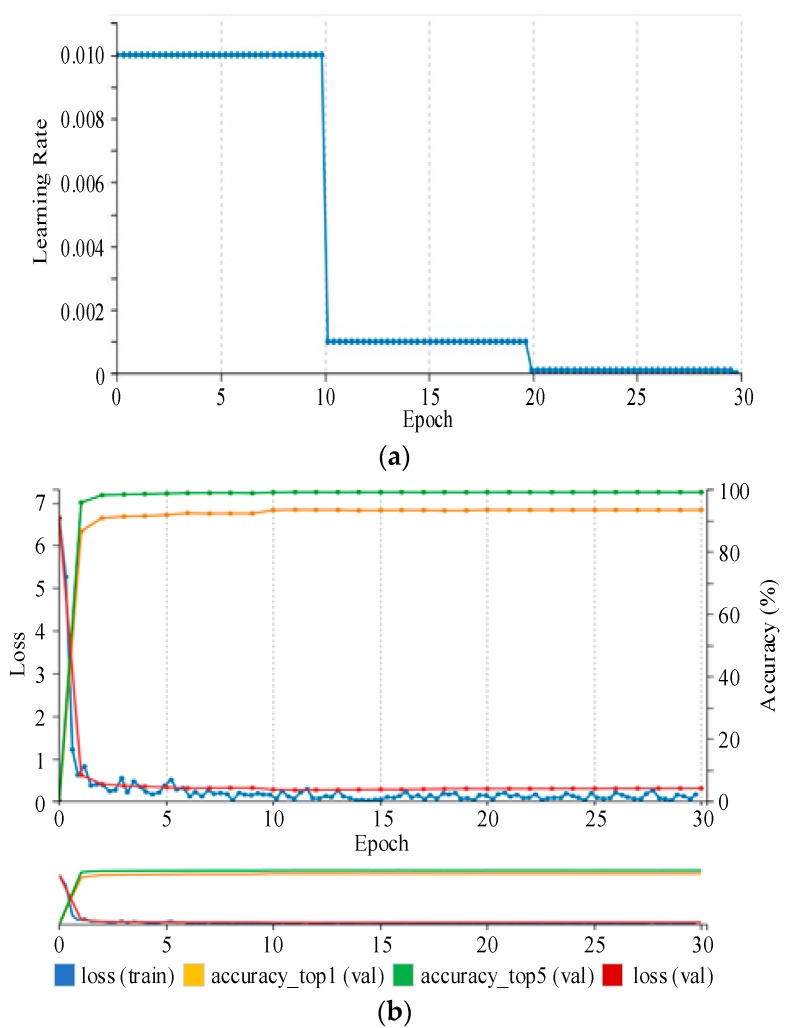
(**a**) Learning rate decay is visualized over training epochs. Here, a step function decay is used, and the learning rate is divided by 10 after one-third and two-thirds of training. (**b**) Training loss, validation loss and accuracy are plotted over training epochs.

An Nvidia DIGITS is used as a second framework that works on top of Caffe, or more precisely, a separate Caffe fork by Nvidia of the official repository. For training, DIGITS provide functionality for on-the-fly data augmentation using random crops with a fixed size, e.g., randomly taking 227 × 227 regions from a 256 × 256 image in our case, and random horizontal flipping. Furthermore, DIGITS visualize learning rate decay as shown in Figure 9. Visualizations of training loss, validation loss, and validation accuracy in an interactive, constantly updating graph make it easy to see whether or not the training is going well. A disadvantage is that, because DIGITS work on a fork of Caffe, new developments of the Caffe repository are merged into it only when the developers of DIGITS decide to do so.

## 5. Performance Evaluation

### 5.1. Dataset

Data from real streets using cameras are collected from seven urban areas in Korea and labelled to create our own dataset of vehicles, yielding over 291,602 labelled vehicle images for vehicles recognition. The labelled images are cropped automatically for classification by using symmetrical filter and split into training, validation and test sets to train and evaluate the neural networks. For the evaluation, we use the rule of thumb according to the Pareto Principle and select 20% as the test set and split the remainder again into an 80% training set and a 20% validation set. A subset of our cropped vehicle dataset which include all the 766 classes and labels will be available at: https://sites.google.com/view/jbnu-selab.

### 5.2. Performance of SqueezeNet and Residual SqueezeNet

We evaluate the performance and properties of SqueezeNet and Residual SqueezeNet trained with vehicle datasets to prove the following hypotheses. First, we show that SqueezeNet recognition is more accurate as compared to AlexNet in terms of classification and more robust as compared to AlexNet and GoogLeNet in terms of speed. The performance of Residual SqueezeNet surpasses that of SqueezeNet in terms of recognition. Second, we analyze the generalization capabilities of the classifier network and changes to the amount of data provided. Lastly, we compressed SqueezeNet and Residual SqueezeNet to less than 5 MB, which is 53 times smaller than AlexNet and 11 times smaller than GoogLeNet in size.

As shown in Table 2, we have trained SqueezeNet model with training samples of 233,280, which contain all 766 classes for 30 epochs and tested the model with 58,322 samples, which contain all the 766 classes. The SqueezeNet model achieved rank-1 and rank-5 accuracies of 94.23% and 99.38% respectively. The training loss is almost negligible, as shown in Table 2. After adding the bypass connections around Fire modules 3, 5, 7, and 9, these modules learn a residual function between input and output. Interestingly, the simple bypass enabled an improvement in accuracy as compared to the architecture without bypass connections. Adding the simple bypass connections yielded an increase of 2.1 percent-age-points in rank-1 accuracy and 0.14 percentage-points in rank-5 accuracy without increasing the model size.

### 5.3. Generalization of New Data and Processing

Evaluation of the generalization capabilities of the model and dataset is important for demonstrating its utility for new applications. We tested SqueezeNet and Residual SqueezeNet in real-time with new video data, which contains 112 vehicles, out of which, 111 are correctly recognized. We also recorded recognition time per vehicle in the video, which was calculated to be 108.81 ms using SqueezeNet, as shown in Table 3. Residual SqueezeNet gives the same accuracy in terms of recognition, but in terms of recognition time, Residual SqueezeNet took 0.44 ms more than SqueezeNet recognition time. However, the recognition capabilities of both models prove that we can use both models in real-time due to their fast processing speed.

### 5.4. Model Size and Number of Parameters

We proposed decreasing the number of parameters by using squeeze layers to decrease the number of input channels by 3 × 3 filters. With the reduction in the number of parameters, the SqueezeNet and Residual SqueezeNet file size is compressed to less than 5 MB, which is 53 times smaller than AlexNet and 11 times smaller than GoogLeNet in size. The number of parameters and the size of the model is summarized in Table 4.

### 5.5. Comparison of the Proposed Method with State-Of-The-Art Methods

Owing to the traditional method-based literatures of vehicle MMR difficult-to-handle large-scale class data, they used fewer vehicle classes for experiments [27,28,29,30]. Thus, it is unrealistic to make exact comparisons. Nevertheless, to provide different views to analyse our work, as did in [31], we list other relevant works’ results as listed in Table 5. 

From it, we can observe that the proposed method achieves a state-of-the-art rank-1 accuracy on the 766 models, which indicates the promising performance of our method for real-world application. Other performance parameters, such as execution time and memory usage were not compared because the most of these methods were based on traditional image processing technology instead of deep neural networks.

For a fair comparison, in this study, we also compared our results with popular deep networks of AlexNet and GoogLeNet, as reported in [26], which contemplated large-scale vehicle models. We have proposed steps towards a more disciplined approach for the design-space exploration of CNNs. Towards this goal, we have presented SqueezeNet and Residual SqueezeNet architectures for extraction of vehicle information such as make, model, and type. The SqueezeNet CNN architecture, which required less than 2% of parameters as compared to AlexNet, surpassed the AlexNet-level architectures by 94.23% in terms of recognition accuracy on our vehicle database. Additionally, with model compression techniques, we are able to compress SqueezeNet and Residual SqueezeNet file size to less than 5 MB. Using SqueezeNet, we were able to pull up the accuracy to 94.23% of rank-1 level, which surpasses the accuracy for AlexNet, which is 93.57%. By adding the bypass connections, we were able to increase the rank-1 accuracy of SqueezeNet by 2.1% and reduce the loss value with the same model size and comparable time cost. The proposed system, when implemented in real time, proves to be very efficient, with an economical time slice of 108.81 ms. Our system is highly reliable for real-time applications given the subsequent decrease in the number of parameters, memory size and computational time. SqueezeNet and Residual SqueezeNet involve considerably smaller architectures and perform very well when it comes to processing time as compared to GoogLeNet and AlexNet. The proposed model is also memory efficient; the total size of the SqueezeNet and Residual SqueezeNet is 4379 KB, which is much smaller compared to the size of GoogLeNet, which is 49,446 KB and that of AlexNet, which is 229 MB. Furthermore, the performances of small architectures have been found to be better than the later models.

In fact, for a given accuracy, we can design different architectures with a different number of parameters and enable them to search for the suitable network architectures that can achieve the given accuracy. By using the approach provided by SqueezeNet, we were able to achieve better accuracy than AlexNet with a lesser number of parameters by selecting the most important parameters that give the best accuracy and neglecting the parameters that have a lower effect on the accuracy. Another advantage of SqueezeNet over AlexNet and GoogLeNet is fewer number of parameters, which translates into a lower probability of overfitting. Hence, it performs better on the test set and is more generalized to the new data as compared to the AlexNet and GoogLeNet.

Our models have been successful to a greater extent in decreasing the vulnerability of such systems in real-time, especially in terms of recognition. The performance of the proposed Residual SqueezeNet models as compared to other state-of-the-art deep learning models is given in Table 6. By our network, the classification time for each vehicle image achieves 109.54 ms, which much faster than AlexNet and GoogLeNet.

### 5.6. Performance of SqueezeNet with Respect to Fire Modules

To evaluate which architecture was best in terms of accuracy, we performed a series of experiments during the course of our study. In one of our experiments, we tried to modify the SqueezeNet in order to know why we selected eight Fire modules. Our experimental results proved that while using SqueezeNet with four Fire modules we achieved 93.60% of rank-1 accuracy and the simultaneous training loss was 0.1367, whilst the rank-5 accuracy achieved was 99.19%. Similarly, for six Fire modules we achieved 93.58% rank-1 accuracy and the training loss was 0.1765 and 99.14% rank-5 accuracy. For nine Fire modules, we achieved 93.56% accuracy with 0.1725 training losses and 99.19% rank-5 accuracy. Finally, with ten Fire modules we achieved 93.38% accuracy with 0.2044 training losses and 99.17% rank-5 accuracy. We found that the best accuracy of 94.23% is achieved with eight Fire modules, as shown in Table 7.

**Figure 10:**
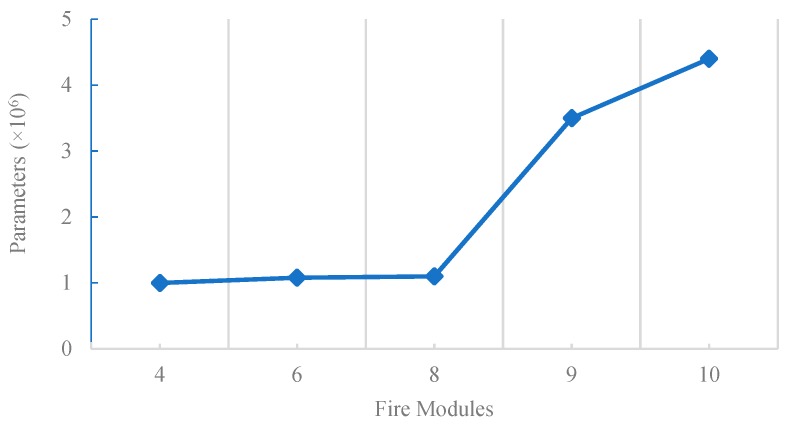
Number of parameters of SqueezeNet with respect to Fire modules.

The graphical trend of the number of parameters with respect to Fire modules is shown in Figure 10. We can see that with the growing number of Fire modules, the size of parameters also increases. As it can be seen, the SqueezeNet with 4, 6 and 8 Fire modules have almost the same parameter number of 1,000,000. When the number of Fire modules exceeds eight, the parameter number increase drastically. This increase results in a corresponding increase in the model size, which, in turn, makes its implementation difficult for use with embedded systems that have a limited memory.

## 6. Conclusions

In this study, an optimized SqueezeNet has been modeled for vehicle MMR. Our contributions are threefold. First, a large-scale dataset for vehicles recognition with over 291,602 images comprising 766 classes has been created and is made publicly available (http://cse.jbnu.ac.kr). Second, a cluster method was proposed to incorporate deep learning techniques to assist faster labeling of a large-scale vehicle make and model dataset. Experimental results demonstrate that our cluster method significantly speeds up the labeling task compared to the manual labeling of each image. Third, we proposed and investigated an approach for a highly robust and real-time automated vehicle MMR based on memory-efficient CNN architectures. Toward this goal, we demonstrated the usefulness of SqueezeNet deep networks employed to address the challenges in VMMR. The SqueezeNet architectures employed enable real-time application use because of compressed model sizes without a considerable change in the recognition accuracy of 96.33% at the rank-1 level. Experimental results have proved the superiority of our proposed system in vehicle MMR. For future work, we plan to further optimize Residual SqueezeNet architecture like adaptive networks to achieve better accuracy and focus on non-frontal vehicle recognition in real-time scenarios. It is also necessary to train our network with larger dataset to handle more vehicle models.

## Figures and Tables

**Table 1:** Examples of label of make, model, and type with their respective class IDs.

ID	Make	Model	Type	ID	Make	Model	Type
1	Hyundai	Grand-Starex	MiniBus	6	Kia	Grand-Carnival	SumoCar
2	Hyundai	Starex-2006-Model	MiniBus	7	Volkswagen	New-CC	Sedan
3	Chevrolet	25 Tons-Cargo-Truck	Truck	8	Samsung	QM3	Sedan
4	Kia	Sorento	SumoCar	9	Hyundai	Porter-2	MiniTruck
5	Hyundai	The-Luxury-Grandeur	Sedan	10	Hyundai	Avante-Hybrid	Sedan

**Table 2:** Performance of SqueezeNet and residual SqueezeNet.

Architecture	No. of Classes	No. of Training Samples	No. of Test Samples	Rank-1 Accuracy	Rank-5 Accuracy	Loss
SqueezeNet	766	233,280	58,322	94.23%	99.38%	0.0516
Proposed Residual SqueezeNet	766	233,280	58,322	96.33%	99.52%	0.0397

**Table 3:** Generalization to new data and processing time.

Architecture	Correctly Recognized Vehicles (out of 112)	Per Vehicle Recognition Time (ms)
SqueezeNet	111	108.81
Proposed Residual SqueezeNet	111	109.25

**Table 4:** Model size and number of parameters.

	AlexNet [23]	GoogLeNet [23]	SqueezeNet	Proposed Residual SqueezeNet
No. of Parameters	59,983,292	6,752,430	1,118,974	1,118,974
Model Size (MB)	229.0	49.4	4.4	4.4

**Table 5:** Reported results of few state-of-the-art methods.

Methods	Classes	No. of Samples	Rank-1 Accuracy (%)	Recognition Time (ms)
He et al. [2]	30	1196	92.47	500.0
Llorca et al. [4]	52	1342	94.00	-
Pearce et al. [27]	74	262	96.00	432.5
Psyllos et al. [28]	11	110	85.00	363.8
Psyllos et al. [29]	10	400	92.00	913.0
Siddiqui et al. [30]	29	6601	94.84	137.9
Fang et al. [31]	281	44,481	98.63	-
Proposed Method	766	291,602	96.33	109.5

**Table 6:** Proposed residual SqueezeNet in comparison with the state-of-the-art deep learning models.

Parameter	AlexNet [23]	GoogleNet [23]	SqueezeNet	Proposed Residual SqueezeNet
Rank-1 Accuracy (%)	93.57	94.31	94.23	96.33
Rank-5 Accuracy (%)	99.02	99.46	99.38	99.52
Loss	0.0835	0.1259	0.0516	0.0397
Parameters (K)	59,983	6752	1118	1118
Size (KB)	229,000	49,446	4379	4379
Classification Time (ms)	294.71	531.40	108.81	109.54

**Table 7:** SqueezeNet performance of models with respect to Fire modules.

No. of Fire Modules	Training Loss	Rank-1 Accuracy (%)	Rank-5 Accuracy (%)
10	0.2044	93.38	99.17
9	0.1725	93.56	99.18
8	0.0516	94.23	99.38
6	0.1765	93.58	99.14
4	0.1367	93.61	99.19
